# Medical Items

**Published:** 1912-08

**Authors:** 


### MEDICAL ITEMS

### AFTER SCARLET FEVER AND MEASLES.

After the acute diseases of childhood there is no remedy that will do more to hasten convalescence than Gray's Glycerine Tonic Comp. Children are particularly responsive to the tonic effects of "Gray's" and it is always gratifying to see the prompt improvement in the appetite, digestion and general nutrition that follows its administration. The palatability and clean bitter taste of "Gray's" make it exceptionally acceptable to children.

---

THERE'S A SURPRISE IN STORE FOR YOU—
and a most agreeable one too, if you have been using chloral and the bromides wherever you wanted to quiet a restless patient or overcome insomnia. The surprise will come when you begin using PASADYNE (Daniel's Concentrated Tincture of Passiflora Incarnata) and find how much more efficient it is than chloral and the bromides, and how free from their dangers and untoward effects. The next time you want to sedate a patient, use PASADYNE and experience the surprise spoken of. A sample bottle will be furnished if application be made to the Laboratory of John B. Daniel, Atlanta, Ga.

---

### THE CHLOROSIS OF YOUNG GIRLS.

To permit the blood stream of chlorotic girls to remain in an impoverished state, is to expose them to more than one peril. Such patients are usually high-school or seminary girls, struggling with duties that tax their every ounce of force. When the break comes, as it almost inevitably will, the physician has on his hands a girl whose recovery takes much time and care. In most instances this could be avoided were the girl put on Cordial of the Extract of Cod Liver Oil Compound (Hagee.)

As a blood-maker and general tissue builder, it is of much value in chlorosis. Not only are the blood corpuscular elements increased in number, but also a noticeable improvement takes place in their quality. Cord. Ext. Ol. Morrhuae Comp. (Hagee) will prove its merit in these cases and its systematic administration over a considerable period of time will save chlorotic girls much of the distress to which they otherwise would be subjected.

